# Predicting Grade of Esophageal Squamous Carcinoma: Can Stretched Exponential Model-Based DWI Perform Better Than Bi-Exponential and Mono-Exponential Model?

**DOI:** 10.3389/fonc.2022.904625

**Published:** 2022-07-14

**Authors:** Hui Yang, Xubo Ge, Xiuzhu Zheng, Xiaoqian Li, Jiang Li, Min Liu, Jianzhong Zhu, Jian Qin

**Affiliations:** ^1^ Department of Radiology, The Second Affiliated Hospital of Shandong First Medical University, Tai’an, China; ^2^ Department of Radiology, The Fourth People’s Hospital of Taian, Tai’an, China

**Keywords:** esophageal squamous carcinoma, magnetic resonance imaging, stretched exponential model, intravoxel incoherent motion, DWI

## Abstract

**Background:**

To evaluate and compare the potential performance of various diffusion parameters obtained from mono-exponential model (MEM)-, bi-exponential model (BEM)-, and stretched exponential model (SEM)-based diffusion-weighted imaging (DWI) in grading of esophageal squamous carcinoma (ESC).

**Methods:**

Eighty-two patients with pathologically confirmed ESC without treatment underwent multi-b-value DWI scan with 13 b values (0~12,00 s/mm^2^). The apparent diffusion coefficient (ADC) deriving from the MEM; the pure molecular diffusion (ADC_slow_), pseudo-diffusion coefficient (ADC_fast_), perfusion, and fraction (f) deriving from the BEM; and the distributed diffusion coefficient (DDC) and water molecular diffusion heterogeneity index (α) deriving from the SEM were calculated and compared between poorly differentiated and well/moderately differentiated ESC, respectively. The prediction parameters and diagnostic efficiency were compared by drawing receiver operating characteristic (ROC) curves.

**Results:**

The ADC, ADC_slow_, ADC_fast_, and DDC in poorly ESC were significantly lower than those in well/moderately differentiated ones. By using only one parameter, ADC_slow_, DDC had the moderate diagnostic efficiency and the areas under the curve (AUC) were 0.758 and 0.813 in differentiating ESC. The DDC had the maximum AUC with sensitivity (88.00%) and specificity (68.42%). Combining ADC with ADC_fast_, ADC_slow_, and DDC and combining ADC_slow_ with ADC_fast_ can provide a higher diagnostic accuracy with AUC ranging from 0.756, 0.771, 0.816, and 0.793, respectively.

**Conclusion:**

Various parameters derived from different DWI models including MEM, BEM, and SEM were potentially helpful in grading ESC. DDC obtained from SEM was the most promising diffusion parameter for predicting the grade of ESC.

## Introduction

Esophageal squamous carcinoma (ESC) is one of the leading reasons of cancer-related mortality (1). Preoperative staging and pathological grading represent important prognostic indicators and determine different treatments. However, preoperative staging of ESC by conventional radiograph can only reflect the morphological changes by visual observation (2). Endoscopy-guided biopsy as a gold standard procedure is widely employed to diagnose ESC as early as possible, but it cannot reflect the grade of whole tumor and is sometimes limited by sampling errors of different observers (3). Magnetic resonance imaging (MRI) without ionizing radiation can provide excellent morphological and functional information for ESC because of its multimodal imaging sequences (4).

Diffusion-weighted imaging (DWI) is a non-invasive functional image sequence in the field of MRI, which uses the movement of water molecules in tissues. The diffusion of water can be quantitatively described by the apparent diffusion coefficient (ADC) (5). The mono-exponential model (MEM), bi-exponential model (BEM), and stretched exponential model (SEM) are all based on standard DWI with varying underlying models and differential governing parameters. Applying multiple models based on DWI protocol, perfusion information could be obtained without the need of intravenous contrast media, which is especially helpful for patients who cannot receive intravenous gadolinium-based contrast media due to severe allergies or compromised renal function. However, as we all know, the ADC featured by a simple mono-exponential decay is obtained from diffusion images with a postulation that the water molecular diffusion is a random motion, which would misestimate the influence of the microcirculation of blood in capillaries and could not reflect the true water diffusion (6). In fact, there are two main aspects that affect the measured diffusion signals in living tissues: one is the motion of water molecules, and the other is the perfusion of blood microcirculation with low b values (less than 200 s/mm^2^), which may lead to an inaccurate estimation of the diffusion. In 1986, Le Bihan et al. (7) by using multi-b-value DWI with a bi-exponential curve fitting firstly described a new imaging technique named intravoxel incoherent motion (IVIM), which has been used to quantitatively assess the microscopic translational motion on MRI. Also, later in 2003, Bennett et al. (8) initially introduced the stretched exponential model, which can assess the diffusion and heterogeneity of living tissues and has been used in several clinical studies (9).

To our knowledge, BEM-based DWI had been used to evaluate the tumor stage and pathological grade of ESC as well as predict treatment response (10). However, there was a lack of research of the SEM. Therefore, the purpose of this study was to investigate the ability and potential additional values of SEM-based DWI in differentiating the pathological grade of ESC.

## Materials and methods

### Patient Population

This study was approved by the ethics committee of our hospital, and informed consent was obtained from each patient. Eighty-six patients with ESC in our hospital from January 2018 to December 2019 were collected in the present study. The inclusion criteria were as follows: (1) MRI plain scans including multi-b-value DWI performed in patients with suspicious ESC by barium study of the gastrointestinal tract or CT examinations, and the tumors were all considered resectable. (2) Before the MRI scans, the patients were not treated with radiation therapy, chemotherapy, or biotherapy. The MRI images showed no artifact, which could affect the diagnosis. (3) All patients underwent surgical resections and confirmed with ESC by pathology ultimately. The exclusion criteria were as follows: (1) The quality of the MR images was poor. (2) The tumor was too small (diameter less than 1 cm) to draw the region of interest (ROI). Finally, three patients with poor multi-b-value DWI scans and one patient with large necrosis of tumor (more than 1/4 quadrant of tumor area) were excluded.

### Magnetic Resonance Imaging

The MRI examinations were performed on a 3T scanner (Discovery 750, GE Healthcare, Chicago, IL, USA) with an eight-channel phase array coil. All the patients were in supine position. The scanning range was centered on ESC and covered the whole tumor. All patients were given breath training before examinations. Routine MRI was acquired with a fast spin echo (FSE) sequence with respiratory gating. Axial T2-weighted images were obtained with TR/TE of 9,230/85 ms (effective), and the slice thickness was 8.0 mm with spacing of 0.5 mm; field of view (FOV) 40 × 40 cm^2^; acquisition matrix, 288 × 256; NEX, 2; and acquisition time, 3 min 14 s. Sagittal fat-saturation T2-weighted images were obtained with TR/TE of 10,909/85 ms (effective), and the slice thickness was 6.0 mm with spacing of 0.5 mm; FOV, 40 × 40 cm^2^; acquisition matrix, 288 × 256; NEX, 2; and acquisition time was 3 min 49 s. The axial and sagittal T2-weighted fat-suppressed images were performed for the localization of ESC so as to plan the multi-b-value DWI scans of tumor. The multi-b-value DWI was performed with the following parameters: repetition time/echo time (TR/TE), 4,500/85 ms; FOV, 24 × 24 cm^2^; acquisition matrix, 128 × 128; slice thickness, 6.0 mm; and spacing, 0.5 mm. Thirteen b values from 0 to 1,200 s/mm^2^ (0, 10, 20, 50, 80, 100, 150, 200, 400, 600, 800, 1,000, 1,200) were used in three diffusion directions, and the number of excitations (NEXs) for each b was 2, 2, 2, 2, 2, 2, 2, 2, 2, 4, 6, 8, and 10. The acquisition time was 10 min 3 s.

### Data Analysis

The DWI original imaging was processed using the Advantage Workstation (ADW 4.6 version, GE, US) and post-processed by Functool Workstation to obtain ADC maps. All MRI examinations were independently processed by two radiologists with 15 and 10 years of experience in reading MR imaging. They evaluated the multi-b-value DWI data and were blinded to histopathological results. The ROIs were placed to cover as much of the solid part of the tumor as possible on three consecutive maximal slices in the axial plane. All parameters were measured twice of each three representative slices, and their average values were calculated for future statistical analysis to reduce the effect of different ROI delineation and measurement by different observers.

### Histopathological Examination

All resected specimen were examined by pathological examinations. The interval between MRI scan and the last surgery was less than 7 days, and these patients did not receive any treatment during the interval. According to the seventh edition of the American Joint Committee on Cancer Stage (AJCC, 7th) (11), the pathological differentiation of ESC was categorized into poorly differentiated, moderately differentiated, and well-differentiated carcinoma.

### Statistical Analysis

The IBM SPSS Statistics 23 software (Armonk, NY) and MedCalc 15.8 (Mariakerke, Belgium) were used for statistical analysis. Quantitative parameters were expressed as the means ± standard deviation. Data were tested for normality analysis using the Kolmogorov–Smirnov test and then with the Levene test for variance homogeneity analysis. Independent *t* test or Mann–Whitney *U* test was used to compare the difference of each parameter between the poorly differentiated group and well/moderately differentiated group. The interobserver agreement and variability were evaluated by ICC and Bland–Altman analysis. Values of the first set of measurement were regarded as the parameters for the tumors when the ICC was more than 0.75 (12). When the ICC was less than 0.75, an average of different measurements of two readers was used as the final result for the subsequent analysis. Receiver operating characteristic (ROC) curve analysis was performed to evaluate the diagnostic performance of each DWI parameter in distinguishing the poorly differentiated group from the well/moderately differentiated group, and the sensitivity and specificity of these parameters were calculated. The area under the curve (AUC) of the ROC curve for the significant parameter was calculated and compared by MedCalc. *P* values of less than 0.05 were considered as statistically significant.

## Results

### Patients and Histology Results

Eighty-two cases of patients with ESC were enrolled in this study, including 49 men and 33 women (age range 42–77 years, median age 54 years). There were 12 cases of well-differentiated ESC, 31 cases of moderately differentiated ESC, 14 cases of mix well/moderately differentiated ESC, and 25 cases of poorly differentiated ESC by histopathological examinations, as well as 7 cases located in the upper esophagus, 35 cases in the middle esophagus, 40 cases in the lower esophagus.

### Interobserver Agreement of Measurements Derived From Different DWI Models

**Figure 1:**
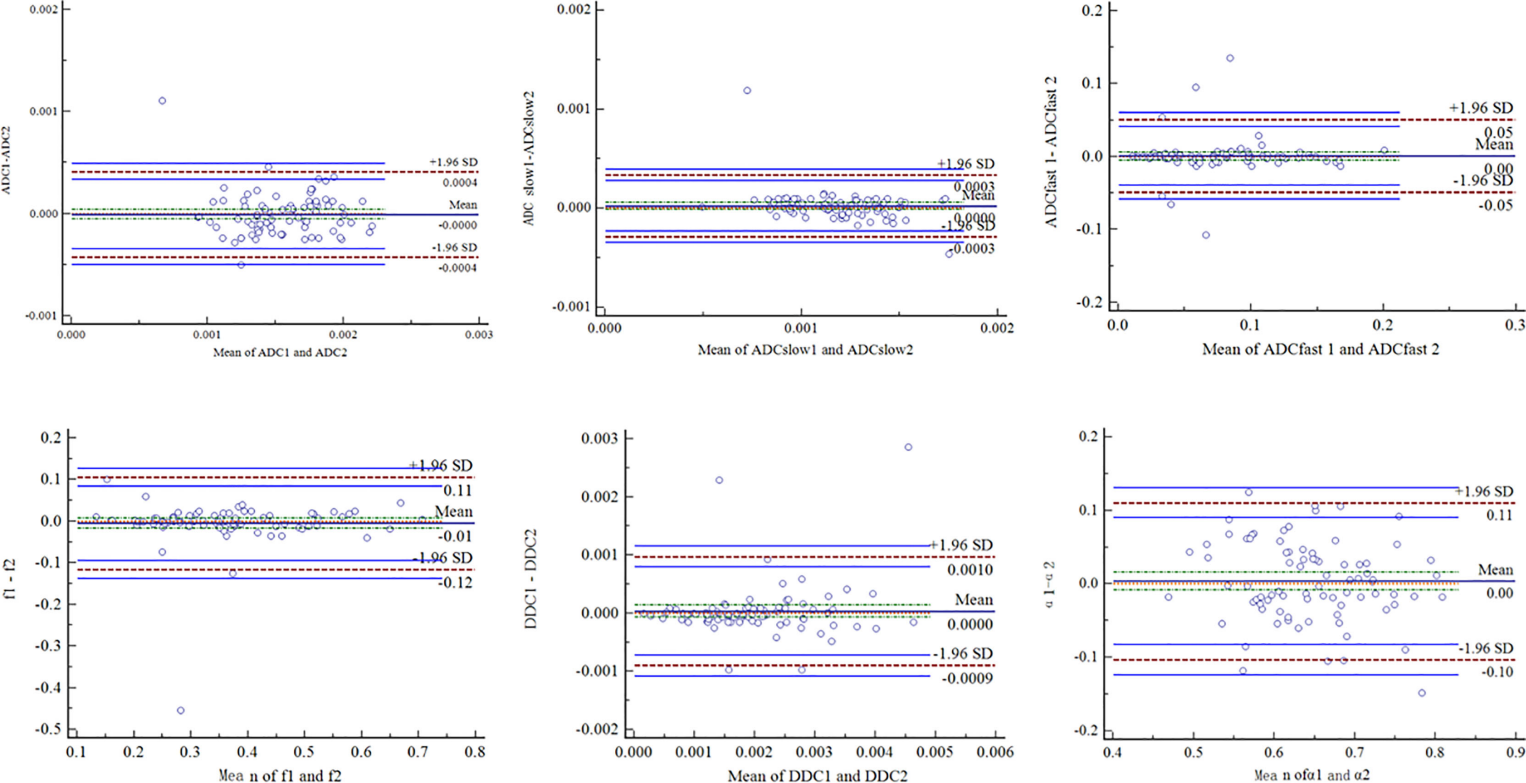
Bland–Altman plots showed interobserver reliability for measurement of different DWI parameters. SD = standard deviation.

The ICC values (**Table 1**) were 0.799 for ADC, 0.804 for ADC_slow_, 0.840 for ADC_fast_, 0.893 for *f*, 0.882 for DDC, and 0.766 for α. The Bland–Altman plots representing the interobserver reproducibility between the two readers are shown in **Figure 1**. The center solid line represents the mean of differences.

**Table 1:** Interobserver reproducibility in the assessment of different DWI parameters.

Parameter	Interclass coefficient correlation	95% confidence interval
ADC	0.799	0.705–0.866
ADC_slow_	0.804	0.713–0.869
ADC_fast_	0.840	0.763–0.894
*f*	0.893	0.854–0.936
DDC	0.882	0.823–0.922
α	0.766	0.659–0.844

ADC, the apparent diffusion coefficient; ADC_slow_, pure molecular diffusion; ADC_fast_, pseudo-diffusion coefficient; DDC, distributed diffusion coefficient; f, fraction; α, water molecular diffusion heterogeneity index.

### Comparisons of Parameters Derived From Various Quantitative DWI Models

**Figure 2:**
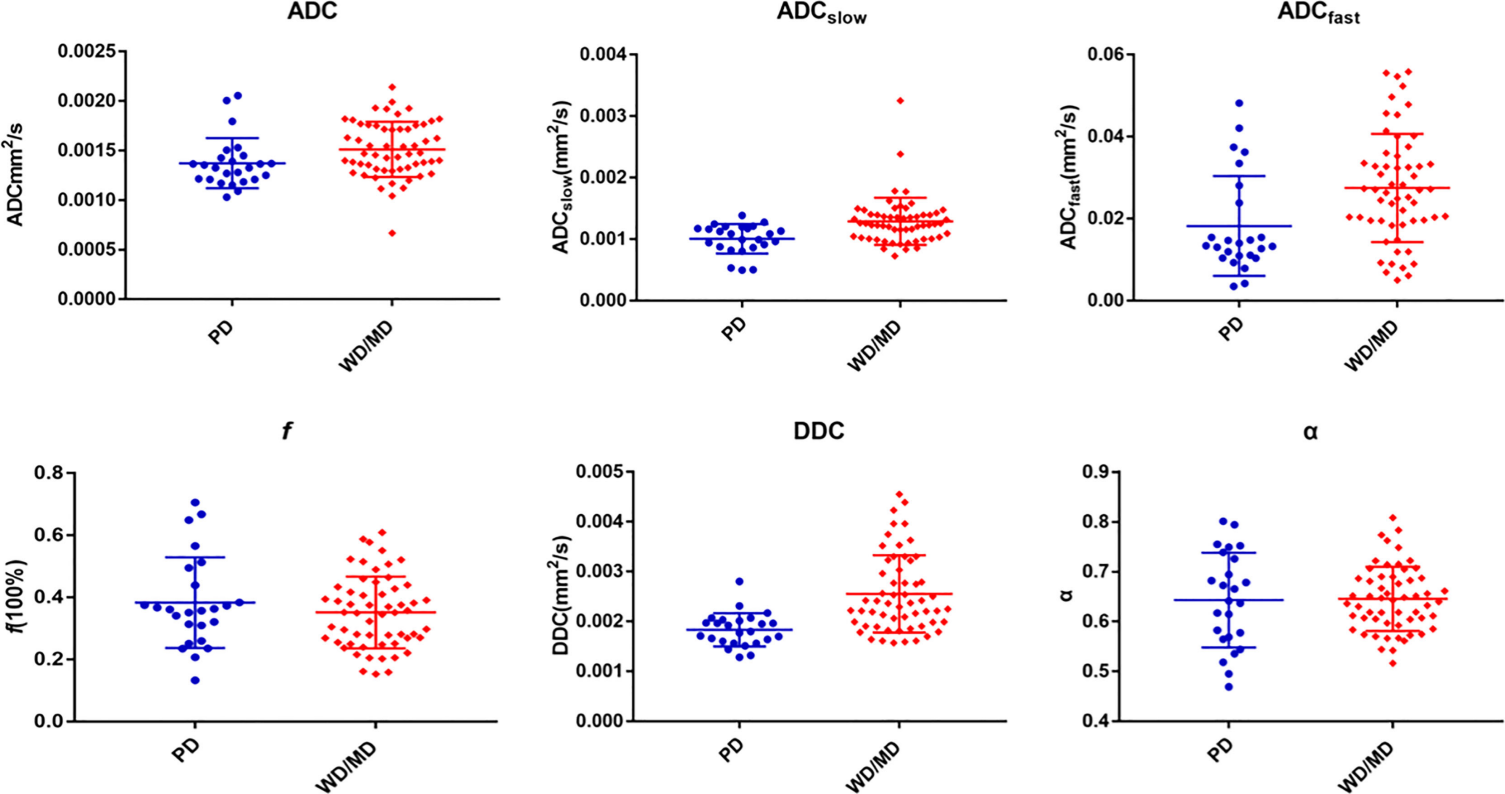
Various parameters derived from different DWI models for comparing PD (poorly differentiated) group with WD/MD (moderately/well-differentiated) group of ESC.

**Figure 3:**
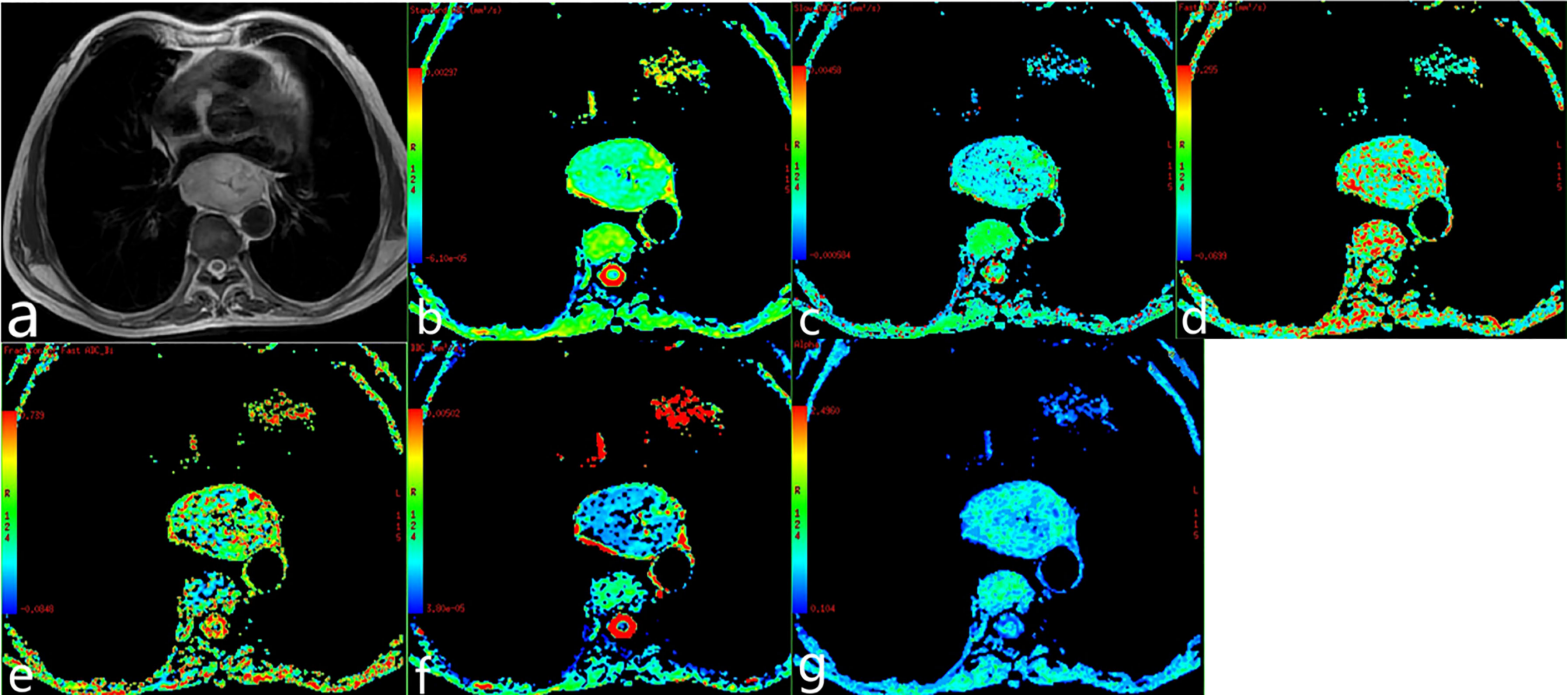
Poorly differentiated esophageal carcinoma of a 62-year-old man. **(A)** T2-weighted image. **(B–G)** ADC map, ADC_slow_ map, ADC_fast_ map, *f* map, DDC map, and α map.

The ADC, ADC_slow_, ADC_fast_, *f*, DDC, and α of different pathologically differentiated ESCs derived from various DWI models are shown in **Table 2** (**Figures 2**, **3**). The results showed that the ADC, ADC_slow_, ADC_fast_, and DDC in the poorly differentiated group were significantly lower than those in the well-/moderately differentiated group (**Table2**). However, there was no significant difference in *f* and α values (**Table 2**).

**Table 2:** Comparison between poorly and well-/moderately differentiated group of different parameters.

	Poorly differentiated group	Well/moderately differentiated group	t/z	P
ADC (×10^-3^ mm^2^/s)	1.372 ± 0.252	1.512 ± 0.277	2.149^a^	0.035
ADC_slow_ (×10^-3^ mm^2^/s)	1.004 ± 0.240	1.287 ± 0.384	-3.702^b^	0.000
ADC_fast_ (×10^-3^ mm^2^/s)	18.197 ± 12.168	27.474 ± 13.212	-2.936^b^	0.003
*f*	0.383 ± 0.145	0.351 ± 0.115	-0.574^b^	0.566
DDC (×10^-3^ mm^2^/s)	1.829 ± 0.334	2.550 ± 0.776	-4.493^b^	0.000
α	0.642 ± 0.094	0.645 ± 0.064	0.140^a^	0.889

a Comparisons were performed by independent t test.

b Comparisons were performed by Mann–Whitney U test ADC, the apparent diffusion coefficient; ADC_slow_, pure molecular diffusion; ADC_fast_, pseudo-diffusion coefficient; DDC, distributed diffusion coefficient; f, fraction; α, water molecular diffusion heterogeneity index.

### Diagnostic Performance of Various Quantitative DWI Models

**Figure 4:**
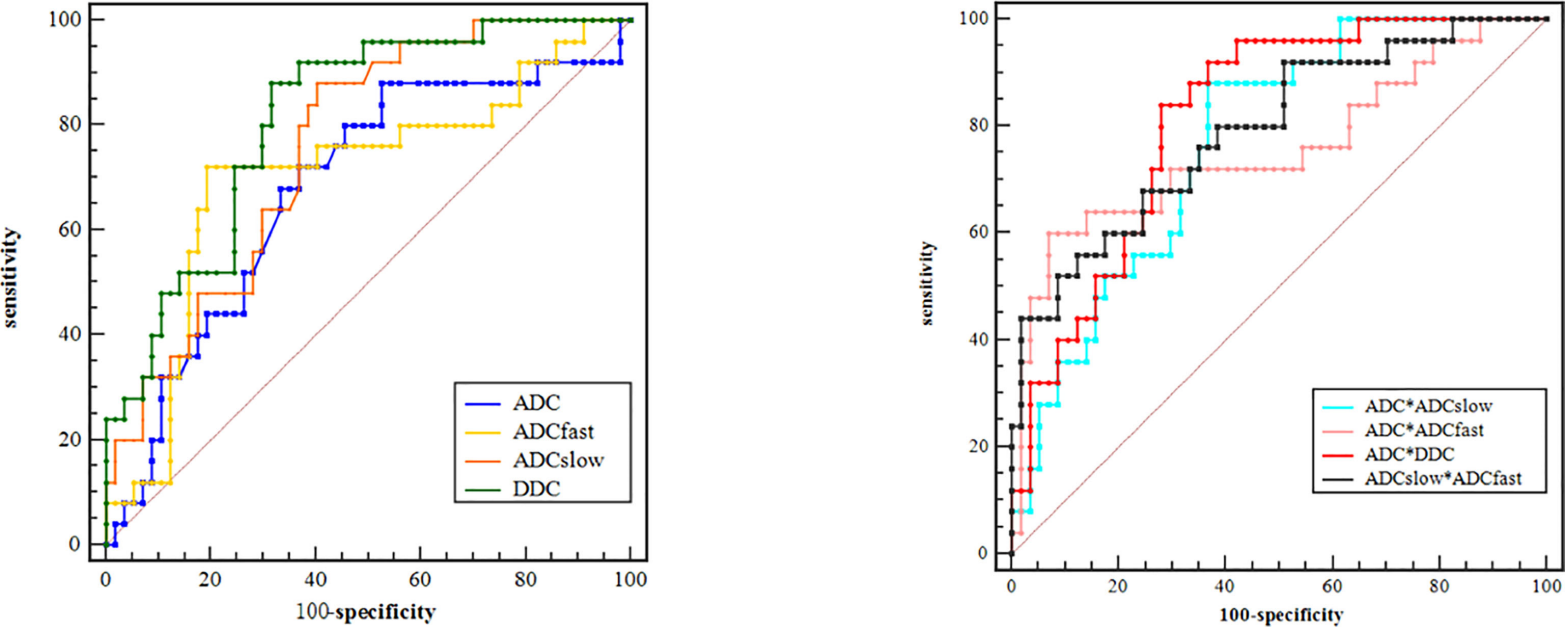
ROC curves of different parameters for identifying PD (poorly differentiated) group with WD/MD (moderately/well-differentiated) group of ESC.

Based on the previous results of the independent *t* test and Mann–Whitney *U* test for comparisons, we performed an ROC analysis of the parameters with significant difference for distinguishing the poorly differently group from the well-/moderately differentiated group, as shown in **Figure 4**. The maximum Youden index, AUC, sensitivity, and specificity are illustrated in **Table 3**. By using only one parameter, ADC_slow_ and DDC had moderate diagnostic efficiency and the areas under the curve were 0.758 and 0.813, respectively. The ROC curves show that DDC had the maximum AUC with sensitivity of 88.00% and specificity of 68.42%; ADC had the minimum AUC. The AUC of DDC was higher than that of ADC, ADC_slow_, and ADC_fast_ but showed no statistical difference (P = 0.110, 0.331, and 0.226). ADC, ADC_slow_, and DDC demonstrated the highest sensitivity (88.00%), but DDC had a higher specificity (68.42%); ADC_fast_ demonstrated the highest specificity with 80.70%. Combining ADC with ADC_fast_, ADC_slow_, DDC, and ADC_slow_ with ADC_fast_ can provide a higher diagnostic accuracy with AUC ranging from 0.756, 0.771, 0.816, to 0.793 respectively (**Table 3**).

**Table 3:** ROC-related parameters in differentiating the poorly and well-/moderately differentiated group of ESC.

Parameters	Maximum Youden index	Area under the curve	Sensitivity (%)	Specificity (%)
ADC	0.354	0.680	88.00	47.37
ADC_slow_	0.476	0.758	88.00	59.65
ADC_fast_	0.527	0.705	70.20	80.70
DDC	0.564	0.813	88.00	68.42
ADC*ADC_fast_	0.529	0.756	60.00	92.98
ADC*ADC_slow_	0.511	0.771	88.00	63.16
ADC*DDC	0.559	0.816	84.00	71.93
ADC_slow_*ADC_fast_	0.437	0.793	56.00	87.72

ADC, the apparent diffusion coefficient; ADC_slow_, pure molecular diffusion; ADC_fast_, pseudo-diffusion coefficient; DDC, distributed diffusion coefficient; f, fraction; α, water molecular diffusion heterogeneity index.

## Discussion

The MRI scans were increasingly used in the staging of gastrointestinal tumors because of their good soft tissue resolution. Applying multiple models based on DWI protocol, perfusion information could be obtained without the need for intravenous contrast media. The main studies of ESC by DWI scans currently were to improve tumor’s early detection and staging accuracy and predict treatment efficacy (13–16). Therefore, the purpose of this study was to investigate the ability and potential additional values of SEM and compare with MEM, BME in differentiating the pathological grade of ESC.

In our present study, the results demonstrated that ADC values were significantly lower in poorly differentiated tumors than in well-/moderately differentiated ones, showing that ADC decreased with a decrease in pathological differentiation, which was consistent with the result of Zhu et al. (10), who also demonstrated that both ADC and ADC_slow_ could assess the tumor cell grade, and the numeric values of the poorly differentiated esophageal carcinoma were lower than the moderately differentiated and well-differentiated groups. Histologically, the decreased value of ADC in poorly differentiated ESC may be related to the following reasons: the fast proliferation of malignant tumor cell, the increase in cell density, the shrinking of the extracellular space, and the increase in the nucleo-cytoplasmic ratio. Huang et al. (17) also found that ADC decreased with the increase in the T stage of ESC, and Mizumachi et al. (18) found that lower ADC values were significantly associated with a higher clinical T stage. Sakurada et al. (13) reported that combining T2WI and DWI obtained detection rates in the T staging of ESC, and they were 33% for T1, 58% for T2, 96% for T3, and 100% for T4, respectively. Kiyohiko Shuto et al. (15) reported that the clinical impact of DWI showed higher sensitivity than PET in predicting postoperative survival for patients with ESC. However, the ADC value was quantified by measuring the mean diffusivity along three orthogonal directions, which was mainly influenced by not only cellularity but also microcirculation. Cellularity and microcirculation would influence ADC measurement in a diametrically opposite direction (19). As we all know, the IVIM can assess the microscopic motion, diffusion, and heterogeneity of living tissues, considering the heterogeneity of the intravoxel diffusion rate and distributed diffusion effect in each voxel in multiple pools of water molecules.

There were few studies focusing on the evaluation of esophageal carcinoma by the IVIM-DWI model. Lei et al. (20) studied the application value of IVIM-DWI in the diagnosis of early esophageal cancer and showed that the *f* value could differentiate between esophageal carcinoma and normal esophagus. Huang et al. (17) found that the IVIM-DWI-derived parameters of D (pure diffusion coefficient) and *f* negatively correlated with the stage of ESC and the D value could distinguish the T1-staged tumor from the normal esophageal wall in detail, which might be probably related to the smooth muscle proliferation and extracellular stroma expansion that blocked the free water diffusion in the progress of ESC. Zheng et al. (21) showed that ADC, D, and *f* increased significantly during concurrent chemoradiotherapy (CRT) and proved that the IVIM-DWI parameters combined with ADC were useful in evaluating treatment and prognosis. Zhu et al. (10) used IVIM and conventional DWI parameters to evaluate the pathologically differentiated grade of esophageal carcinoma and showed that ADC_slow_ and ADC had a significantly higher diagnostic performance than ADC_fast_ and *f*.

As shown in our study, ADC_slow_ representing the pure diffusion was lower than that of ADC and the AUC of ADC_slow_ was higher than that of ADC in distinguishing poorly from well-/moderately differentiated ESC; this was because BEM-based DWI can separate the diffusion and perfusion component from the overall DWI measurement. ADC_slow_ obtained from this model can supply a precise differential diagnosis and reduce the bias by avoiding microcirculation contributions (22). The ADC_slow_ deriving from the BEM model can eliminate the interference of perfusion and maintain the true diffusion, suggesting that ADC_slow_ had a higher diagnostic performance than ADC. It was noted that despite the better diagnostic performance of ADC_slow_, a low value of specificity (59.65%) was present in differentiating poorly from well-/moderately differentiated lesions. This may be related to the pathological heterogeneity of tumor and the overlapping grade of differentiated ESC from the point of the pathology. At the same time, the stability of the MRI parameters may be affected by the surrounding structures such as bone and air.

ADC_fast_ represents a perfusion-related coefficient and reflects microcirculation (22). Our data demonstrated that ADC_fast_ was statistically significant in differentiating the poorly and well-/moderately differentiated lesion. However, the *f* value showed no statistical difference with only a gradually increasing trend from the well-/moderately to poorly differentiated lesion, which was consistent with the research of Zhu et al. (10). However, there were different conclusions for ADC_fast_ in distinguishing the behavior of the tumors in other systems (23, 24). ADC_fast_ was considered proportional to the average blood velocity and capillary segment length (7). The result may be related to the different anatomical blood supply of esophageal segments and the inconsistent capillary length of different differentiated ESCs. Furthermore, because of the low signal-to-noise ratio (SNR) and the limited small b values of measurement, the value of ADC_fast_ may not be reliable and need to be further studied.

SEM is an alternate method that may quantify both tissue heterogeneity and diffusion simultaneously, which was more reliable and reproducible than the MEM- and BEM-based DWI in prior studies (25, 26). The results of our study showed that the DDC values of poorly lesions were significantly lower than those of well-/moderately differentiated ones, and the ROC curves showed that DDC had the maximum AUC, which demonstrated excellent diagnostic performance in differentiating poorly from well-/moderately differentiated lesions. The DDC was considered to be the weighted sum of the continuous distribution of ADCs, and it can provide a more accurate and reliable depiction of tissue diffusion (26, 27). Our results can be explained by the proliferating tumor cells and the increasing nucleus-to-cytoplasm ratio of poorly lesions, which can lead to more intravoxel diffusion heterogeneity. Nevertheless, in our study, the α value was slightly lower in poorly differentiated lesions but showed no statistical difference compared with well/moderately ones. The α value described the deviation of water diffusion from a single exponential decay and was supposed to be related to intravoxel water diffusion heterogeneity, which indicated a numerically low α index (α near 0) representing a high degree of diffusion heterogeneity exhibited as multi-exponential decay, while a numerically high α index (α near 1) represented low intravoxel diffusion heterogeneity approaching mono-exponential decay. Similarly, in the previous studies, Lin et al. (28) did not find a difference between high-grade and low-grade meningiomas, and Wang *et al.* (29) demonstrated that α was not significantly different in the various stages and grades of bladder cancers. However, another former research indicated that the α value can be used to differentiate high-grade and low-grade gliomas with AUC of 0.892 (30). The differences in results suggested that α varied among different types of tumors, which needs further larger cohort studies.

Additionally, the ROC curves were used to distinguish poorly differentiated lesions from well-/moderately differentiated ones. By using only one parameter, DDC had the maximum AUC with sensitivity of 88.00% and specificity of 68.42%, suggesting that it was a reliable diagnostic marker compared with other parameters. Combining ADC with ADC_fast_, ADC_slow_, and DDC and combining ADC_slow_ with ADC_fast_ can provide a higher diagnostic accuracy with AUC ranging from 0.756, 0.771, 0.816 to 0.793, respectively. Therefore, the combination of multiple parameters of different DWI models may have a more powerful diagnostic value.

This study still had several limitations. First, the sample sizes of poorly differentiated ESCs were relatively small and further prospective analysis of a larger number of patients will be needed to validate the present results. Second, the ROIs were placed to cover as much of the solid part of the tumor as possible on three consecutive maximal slices and did not contain the entire volume, which might lead to bias owing to tumor heterogeneity. Therefore, entire tumors should be measured in future research.

In conclusion, various parameters derived from different DWI models including MEM, BEM, and SEM were potentially helpful in grading ESC. DDC obtained from SEM was the most promising diffusion parameter for predicting the grade of ESC.

## Data Availability Statement

The datasets generated during the current study are not publicly available due our research is still ongoing, but they are available from the corresponding author on reasonable request.

## Ethics Statement

This study was approved by the ethics committee of the Second Affiliated Hospital of Shandong First Medical University and informed consent was obtained from each patient. The patients/participants provided their written informed consent to participate in this study. Written informed consent was obtained from the individual(s) for the publication of any potentially identifiable images or data included in this article.

## Author Contributions

HY: conceptualization, writing—original draft preparation. XBG: conceptualization, methodology; XZ: visualization, software; XL: data curation, formal analysis; JL: data curation, methodology; ML: formal analysis, visualization, resources; JZ: supervision, project administration; JQ: supervision, resources, writing—reviewing and editing. All authors contributed to the article and approved the submitted version.

## Funding

The present study was supported by grants from the Academic promotion program of Shandong First Medical University (No. 2019QL017).

## Conflict of Interest

The authors declare that the research was conducted in the absence of any commercial or financial relationships that could be construed as a potential conflict of interest.

## Publisher’s Note

All claims expressed in this article are solely those of the authors and do not necessarily represent those of their affiliated organizations, or those of the publisher, the editors and the reviewers. Any product that may be evaluated in this article, or claim that may be made by its manufacturer, is not guaranteed or endorsed by the publisher.

## Abbreviations

MEM, mono-exponential model; BEM, bi-exponential model; SEM, stretched exponential model; DWI, diffusion-weighted imaging; ESC, esophageal squamous carcinoma; ADC, apparent diffusion coefficient; ADC_slow,_ pure molecular diffusion; ADC_fast_, pseudo-diffusion coefficient; DDC, distributed diffusion coefficient; ROC, receiver operating characteristic; IVIM, intravoxel incoherent motion; ROI, region of interest; TR/TE, repetition time/echo time; NEXs, number of excitations; AUC, area under the curve; SNR, signal-to-noise ratio.
